# Tissue-specific metabolite profiling and quantitative analysis of ginsenosides in *Panax quinquefolium* using laser microdissection and liquid chromatography–quadrupole/time of flight-mass spectrometry

**DOI:** 10.1186/s13065-015-0141-0

**Published:** 2015-12-09

**Authors:** Yujie Chen, Liang Xu, Yuancen Zhao, Zhongzhen Zhao, Hubiao Chen, Tao Yi, Minjian Qin, Zhitao Liang

**Affiliations:** School of Chinese Medicine, Hong Kong Baptist University, Kowloon, Hong Kong Special Administrative Region People’s Republic of China; Department of Resources Science of Traditional Chinese Medicines, State Key Laboratory of Modern Chinese Medicines, College of Traditional Chinese Medicines, China Pharmaceutical University, Tongjiaxiang-24, Gulou District, Nanjing, 210009 People’s Republic of China; School of Pharmacy, Liaoning University of Traditional Chinese Medicine, Dalian, China

**Keywords:** Ginsenosides, *Panax quinquefolium* L., Tissue-specific, Laser microdissection, UHPLC-Q/TOF–MS

## Abstract

**Background:**

The root of *Panax quinquefolium* L., famous as American ginseng all over the world, is one of the most widely-used medicinal or edible materials. Ginsenosides are recognized as the main bioactive chemical components responsible for various functions of American ginseng. In this study, tissue-specific chemicals of *P*. *quinquefolium* were analyzed by laser microdissection and ultra-high performance liquid chromatography- quadrupole/time-of-flight-mass spectrometry (UHPLC-Q/TOF–MS) to elucidate the distribution pattern of ginsenosides in tissues. The contents of ginsenosides in various tissues were also compared.

**Results:**

A total of 34 peaks were identified or temporarily identified in the chromatograms of tissue extractions. The cork, primary xylem or cortex contained higher contents of ginsenosides than phloem, secondary xylem and cambium. Thus, it would be reasonable to deduce that the ratio of total areas of cork, primary xylem and the cortex to the area of the whole transection could help to judge the quality of American ginseng by microscopic characteristics.

**Conclusion:**

This study sheds new light on the role of microscopic research in quality evaluation, and provides useful information for probing the biochemical pathways of ginsenosides.

**Graphical abstract:**
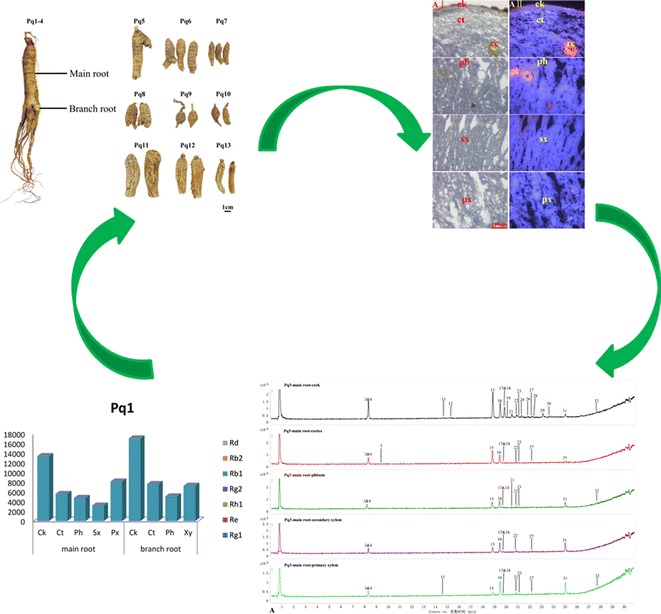
.

## Background

Microscopic authentication refers to examine the structure, cell and internal features of herbal medicines using a microscope and its derivatives. It has been recorded in many Pharmacopoeias as an authentication method, such as Chinese Pharmacopoeia, United States Pharmacopeia, European Pharmacopoeia, British Pharmacopoeia, Japanese Pharmacopoeia, and Korean Pharmacopoeia. Distinctly, microscopic authentication has been commonly used in the authentication of herbal medicines. As we know, the secondary metabolites of herbal medicine contribute to its effects. Nevertheless, the normal microscopic identification cannot provide the useful information of secondary metabolites in different herbal materials directly. Thus, microscopic method can identify the source species but not evaluate the quality of herbal medicines.

By using techniques of anatomy and histochemistry, some studies have demonstrated that there is a close relationship between microscopic characteristics and active components of herbal medicines. For example, the histochemical techniques and phytochemical methods have been applied in the distribution and accumulation of active components in *Sinomenium acutum*, *Aloe vera* var. *chinensis*, *Gynostemma pentaphyllum*, *Dioscorea zingiberensis* and *Macrocarpium officinacle* [1–5]. However, these studies used routine chemical reactions and thus the distribution of the detailed active components could not be identified. Moreover, those agents usually have poor specificity, which leads to the increase of false positive results. Also, it is noteworthy that these investigations lacked objective data and had not been validated by other methods yet. Recently, the combination of fluorescence microscopy, laser microdissection (LMD), and ultra-high performance liquid chromatography-quadrupole/time-of-flight-mass spectrometry (UHPLC-Q/TOF–MS) has been successfully applied to explore the distribution pattern of secondary metabolites among different tissues from several Chinese medicinal materials (CMMs) [6–11]. This method can obtain the exact quantitative and qualitative data to profile the chemicals in tissues and cells of medicinal materials.

American ginseng, the root of *Panax quinquefolium* L., is one of the most recognized herbal medicines all over the world. Also, American ginseng has become popular in oriental countries as dietary health supplements or additives to foods and beverages [12]. In the herbal markets, various specifications or grades of American ginseng can be found, including main root, rootlet and fibrous root. Production area also affects the grade or price of the commercial medicine. As we know, American ginseng contains the major bioactive triterpene saponins named ginsenosides, such as ginsenosides Rg_1_, 20(*S*)-Rg_2_, Re, 20(*S*)-Rh_1_, Rb_1_, Rb_2_ and Rd, which possess a wide range of pharmacological effects, including cardiovascular, anti-diabetic, anti-inflammatory and anti-tumor properties [13–16].

To evaluate the quality of American ginseng, a number of analytical methods to determine the total ginsenoside content or the target compounds have been developed [17–19]. However, few of them focus on the distribution rules of ginsenosides among tissues or detect the relationship of the quality and the microscopic characteristics. Until now, ginsenosides in the rhizome and root of *P. ginseng* Meyer has already been located: the cork contained more kinds of ginsenosides than did the cortex, phloem, xylem and resin canals [8]. But whether this rule applies to *P. quinquefolium* or not still waits to be found out. Analyzing the distribution of ginsenosides in different anatomical structures will establish the relationship between microscopic features and active components. Then the microscopic features used for the quality evaluation and classification of different specifications or grades of American ginseng can be validated or clarified.

In this study, fluorescence microscopy, LMD and UHPLC-Q/TOF–MS were used to analyze and compare the spatial chemical profiles of various tissues from *P. quinquefolium* to correlate the relationship between microscopic features and active components for the quality evaluation of American ginseng, shedding new light on the role of microscopic research in quality evaluation.

## Results and discussion

### Microscopic examination and dissection by LMD

**Fig. 1:**
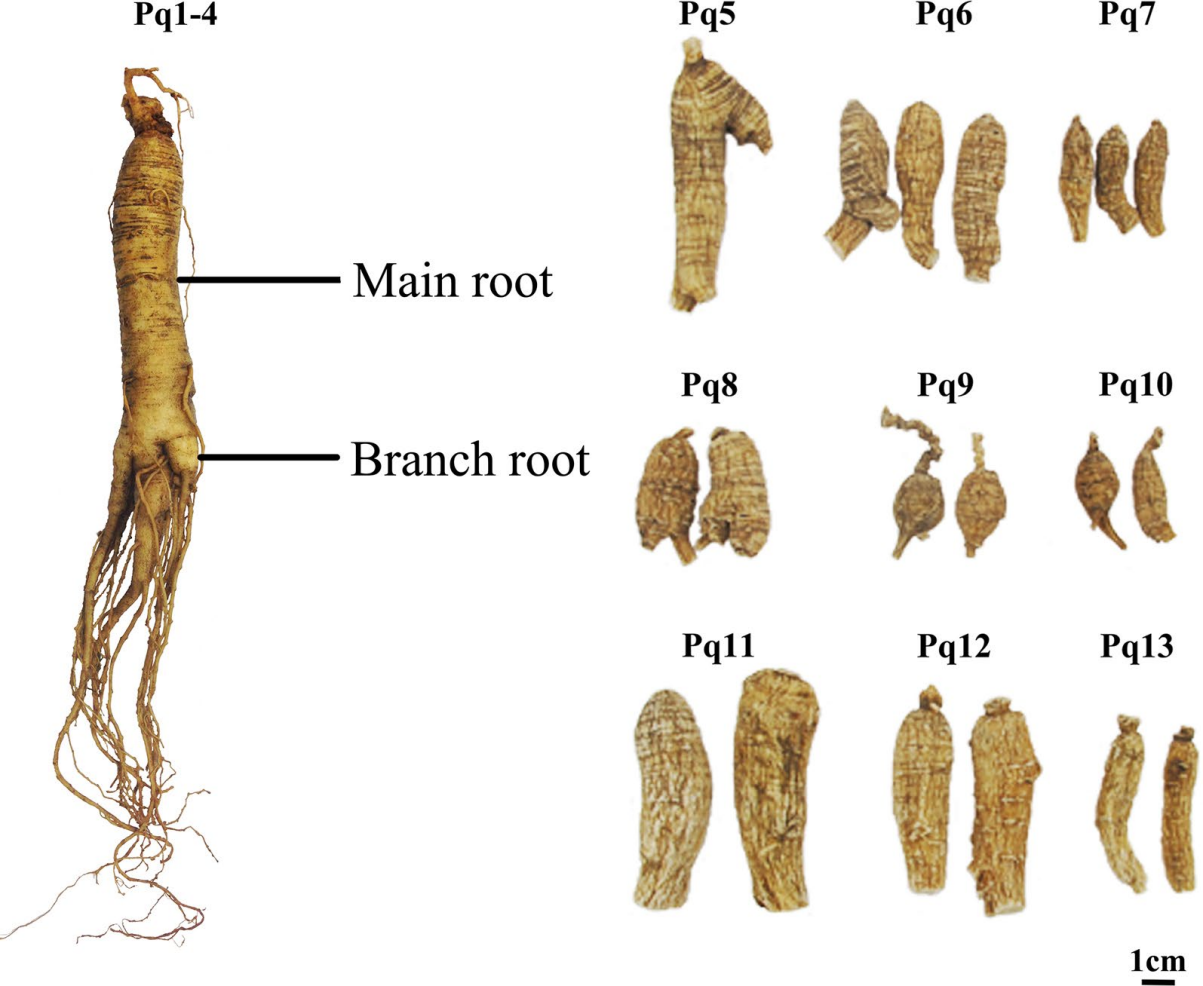
Morphological features of *Panax quinquefolium* materials

**Fig. 2:**
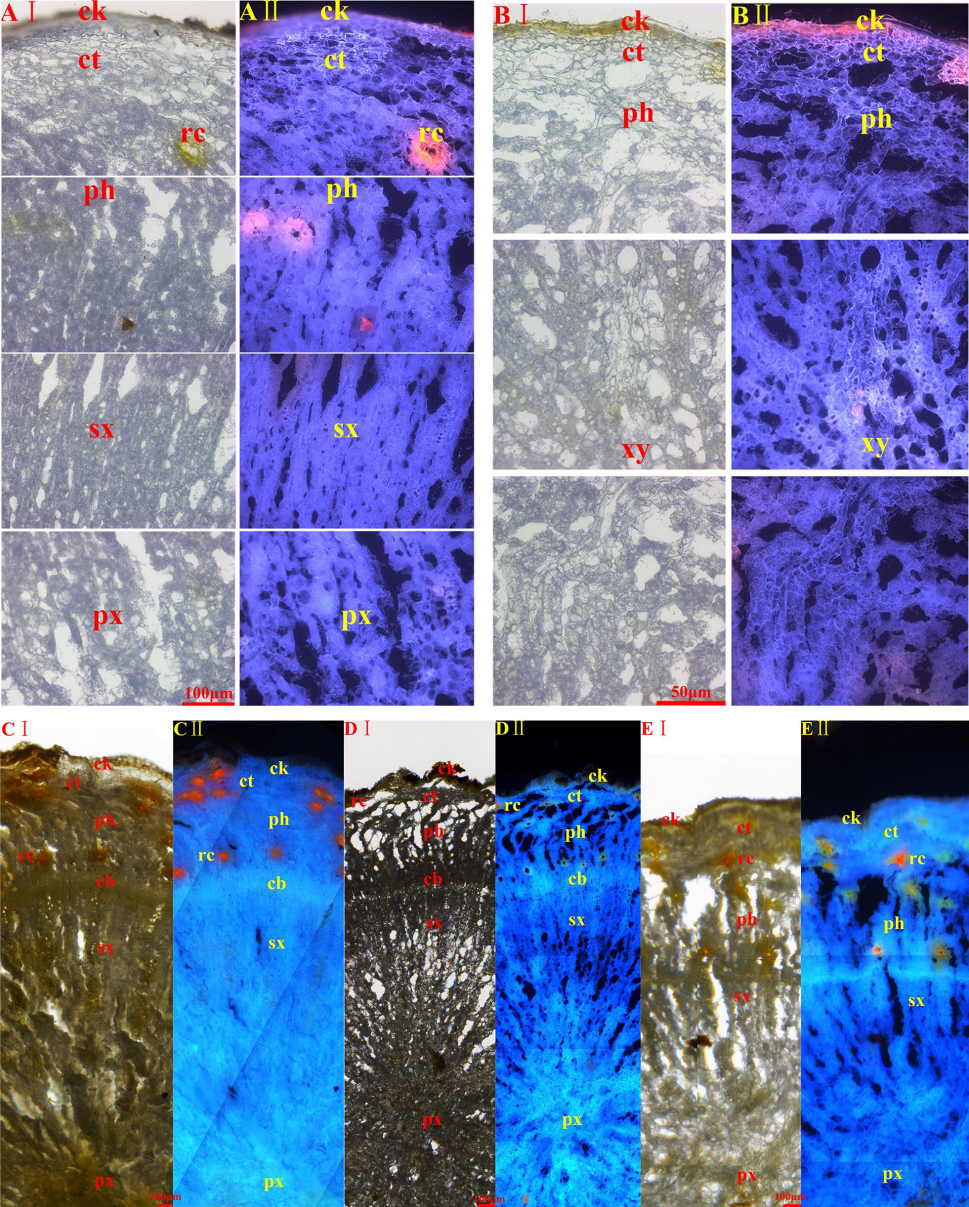
Microscopic characteristics of *P. quinquefolium.* I Under normal light microscope, II under fluorescence mode with dichromatic mirror. **a**, **b** represented the main root and branch root of Pq1; **c**–**e** represented Pq6, Pq8 and Pq10 respectively. *ck* cork, *ct* cortex, *ph* phloem, *rc* resin canals, *cb* cambium, *xy* xylem, *sx* secondary xylem, *px* primary xylem, *pt* pith

In this study, four fresh *P*. *quinquefolium* samples (Pq1–4) and nine dried commercial samples were collected for analysis (see Table 1; Fig. 1). As shown under the normal light and fluorescence mode (see Fig. 2), the transverse section of American ginseng was comprised of cork, cortex, phloem, cambium and xylem. The cork was consisted of several rows of densely-arranged flat cells. Red fluorescence was emitted from the cork while blue color was shown in other tissues. Cortex was narrow. Cracks could be seen in phloem. Resin ducts with orange red fluorescence were scattered in the cortex and phloem. Cambium was arranged in a ring, showing strong florescence. Xylem was broad, usually differentiated into primary xylem with strong florescence and secondary xylem with common florescence. Since our study on localization of ginsenosides in the rhizome and root of *P. ginseng* illustrated that the resin ducts contained few ginsenosides, the resin ducts of *P. quinquefolium* samples were not examined here. The cork, cortex, phloem, secondary xylem and primary xylem were dissected from the main roots of Pq1–4 and Pq5–13. For the branch roots of Pq1–4, the xylem was hardly seen differentiation, and was thus examined as a whole. Compared with other samples, the cambium in the cross sections of Pq6 and Pq8 was obvious with relative more layers of cells, hence, the cambium of Pq6 and Pq8 were also investigated. Therefore, various tissues possessed different features and could be recognized under fluorescence mode. According to previous reports [6–8], the size of about 2,500,000 and 1,000,000 μm^2^ of each separated tissues of fresh and dried materials were dissected by LMD respectively which could detect the chemicals containing in tissues.

**Table 1:** Information of commercial samples of *Panax quinquefolium* materials

Sample no.	Commercial name	Specification	Harvest time	Harvest place
Pq1	American ginseng	–	September 12th, 2014	Cultivation in Mulin County, Mudanjiang City, Heilongjiang Province, China
Pq2	American ginseng	–	September 12th, 2014	Cultivation in Mulin County, Mudanjiang City, Heilongjiang Province, China
Pq3	American ginseng	–	September 12th, 2014	Cultivation in Mulin County, Mudanjiang City, Heilongjiang Province, China
Pq4	American ginseng	–	September 12th, 2014	Cultivation in Mulin County, Mudanjiang City, Heilongjiang Province, China
Pq5	Wild-mountain pao-shen no. 1	HK$ 66,137.57/1000 g	–	Wildlife in America
Pq6	Wild-mountain small pao-shen no. 3.5	HK$ 34,391.53/1000 g	–	Wildlife in America
Pq7	Wild-mountain small and rouond pao-shen	HK$ 25,873.02/1000 g	–	Wildlife in America
Pq8	Wild-mountain pao-mian no. 3.5	HK$ 76,190.48/1000 g	–	Wildlife in America
Pq9	Wild-mountain pao-mian no. 4	HK$ 52,645.5/1000 g	–	Wildlife in America
Pq10	Wild-mountain small and rouond pao-mian	HK$ 44,973.54/1000 g	–	Wildlife in America
Pq11	Cultivated big-branch Pao-shen	HK$ 1534.39/1000 g	–	Cultivation in Canada
Pq12	Cultivated middle-branch Pao-shen	HK$ 1428.57/1000 g	–	Cultivation in Canada
Pq13	Cultivated shen no. 4	HK$ 1111.11/1000 g	–	Cultivation in Canada

### Tissue-specific chemical profiles

**Fig. 3:**
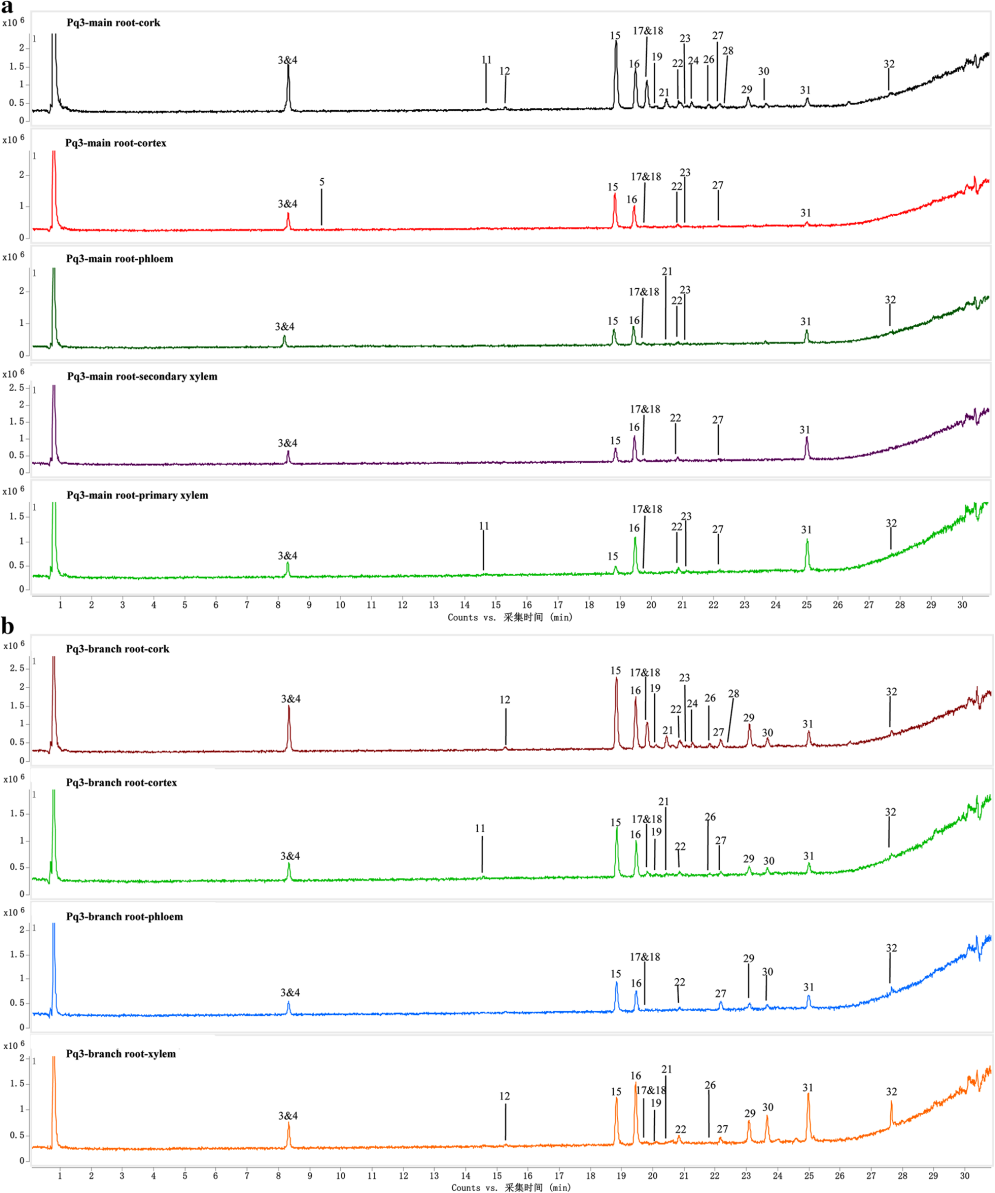
The total ions current (TIC) chromatograms of microdissected tissues from main root (**a**) and branch root (**b**) of *P. quinquefolium* samples. The peak numbers referred to Table 2

**Fig. 4:**
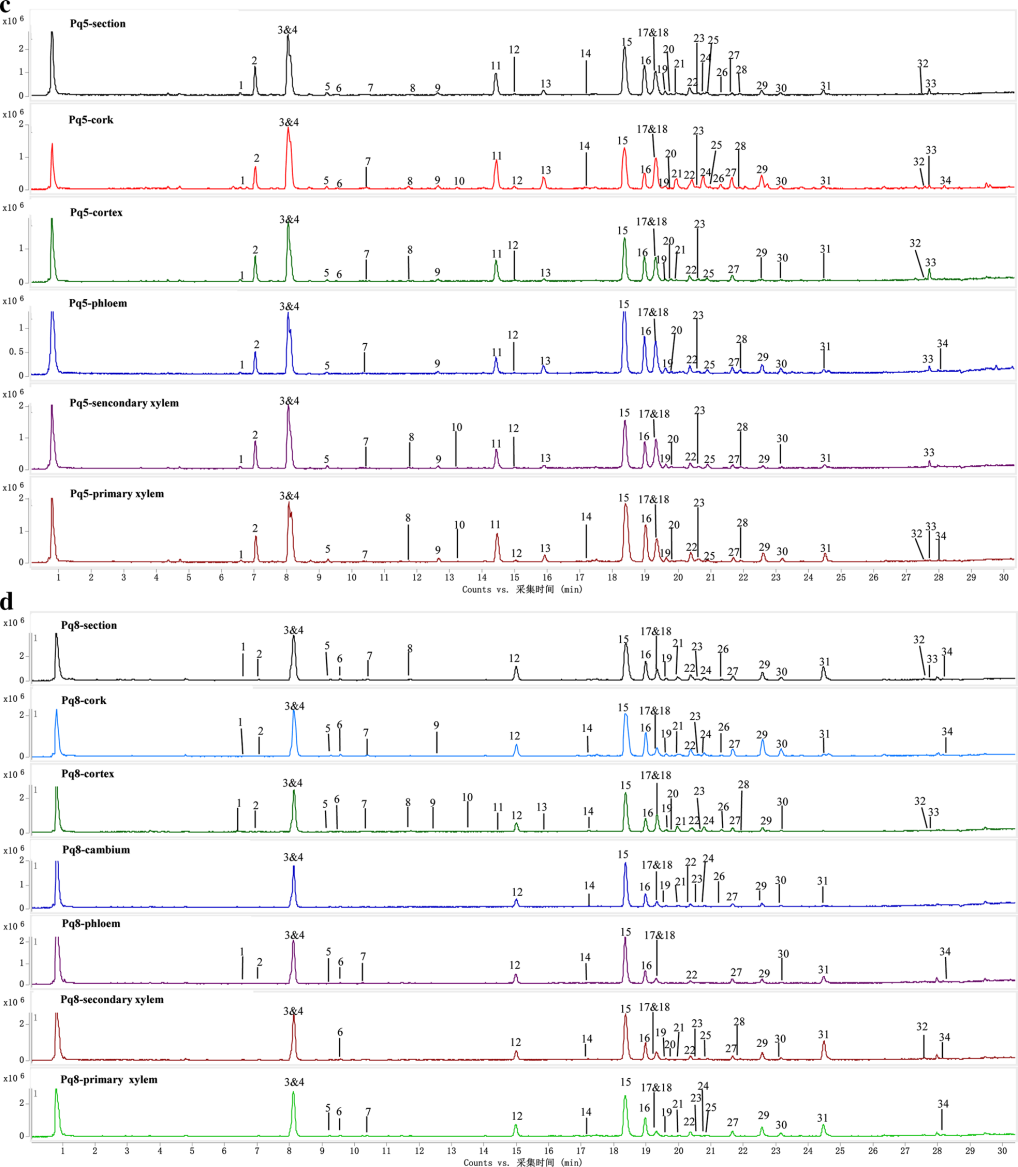
The total ions current (TIC) chromatograms of microdissected tissues from *P. quinquefolium* samples of Pq5 (**c**) and Pq8 (**d**). The peak numbers referred to Table 2

By UHPLC-Q/TOF–MS technique, tissue-specific chemical profiles of each sample were obtained as total ion chromatograms (see Figs. 3, 4). A total of 34 peaks were detected in all the tissue extractions. By comparing retention times, accurate mass weights, and mass ions with the reference compounds, six peaks (Peaks 3, 4, 14, 15, 23, 29) were unambiguously identified as ginsenosides Rg_1_, Re, 20(S)-Rg_2_, 20(S)-Rb_1_, Rb_2_ and Rd. By matching those data with the components reported in the literature, 25 compounds were tentatively authenticated [12, 20–24]. The identification result is shown in Table 2.

**Table 2:** Compounds identified from tissue extractions of *Panax quinquefolium* samples

Peak no.	Identity	t_R_ (min)	Molecular formular	[M−H]^+^			[M−H+HCOOH]^+^ (mass accuracy, ppm)	Fragments of [M−H]^+^ (*m*/*z*)
				Mean measured mass (Da)	Theoretical exact mass (Da)	Mass accuracy (ppm)		
1	20-Glc-G-Rf	6.58	C_48_H_82_O_19_	961.5522	961.5378	14.98	1007.5578	799.5047 [M−H−Glc]^−^
2	Notoginsenoside R_1_	7.04	C_47_H_80_O_18_	931.5186	931.5278	−9.88	977.5465	799.5026 [M−H−Xyl]^−^ 637.4285[M−H−Glc−Xyl]^−^;
3	G-Rg_1_^a^	8.04	C_42_H_72_O_14_	799.4975	799.4849	15.76	845.5026	637.4360 [M−H−Glc] ^−^ 475.3785 [M−H−2Glc]^−^
4	G-Re^a^	8.12	C_48_H_82_O_18_	945.5548	945.5428	12.69	991.5630	799.4935 [M−H−Rha]^−^ 783.5029 [M−H−Glc]^−^ 637.4407 [M−H−Rha−Glc] ^–^
5	Malonyl-G-Rg_1_	9.25	C_45_H_74_O_17_	885.5082	885.4853	25.86	–	841.3240 [M−H−CO_2_]^−^
6	Malonyl-G-Re isomer	9.56	C_51_H_84_O_21_	1031.5547	1031.5432	11.15	–	987.5678[M−H−CO_2_]^−^
7	Malonyl-G-Re	10.32	C_51_H_84_O_21_	1031.5549	1031.5432	11.34	–	987.5644[M−H−CO_2_]^−^
8	Floralquinquenoside B	11.73	C_42_H_72_O_15_	815.4884	815.4793	11.16	–	637.4381[M−H−Rha−CH_3_OH]^−^
9	Floralquinquenoside D	12.65	C_42_H_72_O_15_	815.4882	815.4793	10.91	861.5002	653.4360 [M−H−Glc]^−^
10	Unknown	13.26	–	961.5559	–	–	1007.5580	–
11	Notoginsenoside Rw_2_	14.43	C_41_H_70_O_14_	785.4780	785.4687	11.84	831.4871	653.4361 [M−H−Xyl]^−^ 491.3674 [M−H−Xyl−Glc]^−^
12	Pseudoginsenoside F_11_	14.99	C_42_H_72_O_14_	799.4831	799.4844	−1.63	845.5015	653.4385 [M−H−Rha]^−^
13	Notoginsenoside R_2_	15.89	C_41_H_70_O_13_	769.4573	769.4738	−21.44	815.4730	637.4392 [M−H−Xyl]^−^ 475.3839 [M−H− Xyl−Glc]^−^
14	20 *(S)*-G-Rg_2_^a^	17.23	C_42_H_72_O_13_	783.5029	783.4900	16.46	829.5054	637.4394 [M−H−Rha]^−^ 475.3734 [M−H−Rha−Glc]^−^
15	G-Rb_1_^a^	18.38	C_54_H_92_O_23_	1107.6097	1107.5957	12.64	–	945.5552[M−H−Glc]^−^ 783.5012 [M−H−2Glc] ^−^
16	Malonyl-G-Rb_1_	18.99	C_57_H_94_O_26_	1193.6113	1193.5961	12.73	–	1149.6201[M−H−CO_2_]^−^
17	G-Ro	19.33	C_48_H_76_O_19_	955.5077	955.4908	17.69	–	793.2586[M−H−Glc]^−^
18	G-Rc	19.34	C_53_H_90_O_22_	1077.5730	1077.5871	−13.08	–	945.5660 [M−H−Araf]^−^ 783.4980 [M−H−Araf −Glc]^−^
19	Malonyl-G-Rb_1_ isomer I	19.63	C_57_H_94_O_26_	1193.6142	1193.5961	15.16	–	1149.6185[M−H−CO_2_]^−^
20	Unknown	19.80	–	1087.5461	–	–	–	
21	Malonyl-G-Ra_2_	19.97	C_56_H_92_O_25_	1163.5993	1163.5855	11.86	–	1119.6041[M−H−CO_2_]^−^
22	Malonyl-G-Rb_1_ isomer II	20.38	C_57_H_94_O_26_	1193.6101	1193.5961	11.73	–	1149.6192[M−H−CO_2_]^−^
23	G-Rb_2_^a^	20.47	C_53_H_90_O_22_	1077.5683	1077.5851	−15.59	1123.6337	945.5674 [M−H−Arap]^−^
24	G-Rb_3_	20.79	C_53_H_90_O_22_	1077.5977	1077.5851	11.69	1123.6637	945.5587 [M−H−Xyl]^−^ 915.5474 [M−H−Glc]^−^
25	Unknown	20.91	–	1119.6015	–	–	–	925.4844
26	Ma- Rb_2_/Rb_3_ isomer	21.34	C_56_H_92_O_25_	1163.5992	1163.5849	12.29	–	1119.6007[M−H−CO_2_]^−^
27	*O*-acetyl-G-Rb_1_	21.68	C_56_H_94_O_24_	1149.6198	1149.6062	11.83	1195.6270	1107.6067 [M−H−Acetyl]^−^ 945.5466 [M−H−Acetyl−Glc]^−^
28	Zingibroside R_1_	21.92	C_42_H_65_O_14_	793.4479	793.4374	13.23	–	631.3332[M−H−Glc]^−^
29	G-Rd^a^	22.59	C_48_H_82_O_18_	945.5548	945.5428	12.69	991.5613	783.4985 [M−H−Glc]^−^ 621.4432 [M−H−2Glc]^−^
30	Malonyl-G-Rd	23.18	C_51_H_84_O_21_	1031.5614	1031.5432	17.64	–	987.5682[M−H−CO_2_]^−^
31	G-Rd isomer	24.49	–	945.5543	945.5428	12.16	991.5069	783.4985 [M−H−Glc]^−^ 621.4432 [M−H−2Glc]^−^
32	20 *(S)*-G-Rg_3_	27.55	C_42_H_72_O_13_	783.4978	783.4900	9.96	829.5057	621.4375 [M−H−Glc]^−^ 459.4088 [M−H−2Glc]^−^
33	Chikusetsusaponin IVa	27.69	C_42_H_66_O_14_	793.4367	793.4380	−1.64	–	–
34	20 *(R)*-G-Rg_3_	28.14	C_42_H_72_O_13_	783.4982	783.4900	10.47	829.5065	621.4375 [M−H−Glc]^−^ 459.3964 [M−H−2Glc]^−^

*G* ginsenoside, *Glc* *β*-d-glucopyranosyl, *Rha* *α*-l-rhamnopyranosyl, *Xyl* *β*-d-xylopyranosyl, *Araf* *α*-l-arabinofuranosyl, *Arap* *α*-l-arabinopyranosyl

^a^Identified with chemical marker

As seen from Figs. 3, 4, the distribution differences of gensenosides in various tissues from American ginseng were not as distinct as Asian ginseng [8]. The cork extractions usually had the most peaks (20–34 peaks). The cortex and primary xylem took the second place, namely 11–31 peaks and 12–30 peaks respectively. The secondary xylem (9–28 peaks), phloem (11–27 peaks) and cambium (24 peaks for Pq6, 18 peaks for Pq8) possessed the least peaks. For example, the cork, cortex, phloem, secondary xylem and primary xylem of Pq1 showed 34, 29, 29, 28 and 30 peaks separately. The tissues above of Pq7 had 32, 19, 14, 19 and 21 peaks respectively. Thus, the cork, primary xylem and cortex possessed the most kinds of saponin compounds.

For most samples, the areas of Peaks 21–30 in the cork were larger than those in other tissues. Peaks 21–30 represented compounds with medium or low polarity, which might be concerned with the protection function of the cork. In the xylem, especially the primary xylem, the areas of Peaks 17–31 were larger than those in cortex, phloem and cambium, which might be relevant with the lignification, suberification and the channel function of xylem cells.

### Quantification of ginsenosides in various tissues

**Fig. 5:**
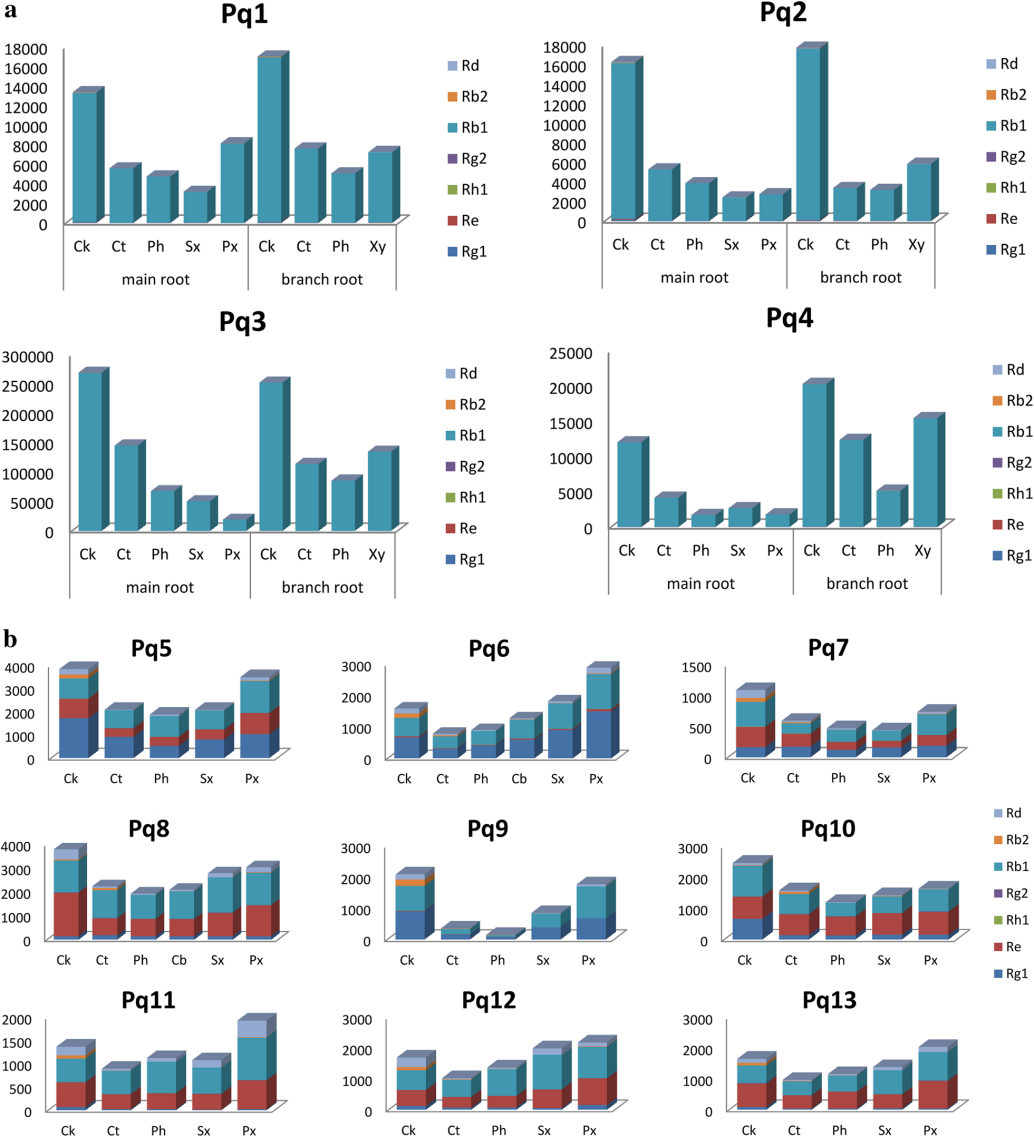
Contents of ginsenosides in different tissues of Pq1-4 (**a**) and Pq5-13 (**b**). *Ck* cork, *Ct* cortex, *Ph* phloem, *Cb* cambium, *Sx* secondary xylem, *Px* primary xylem

Ginsenosides Rg_1_, Re, Rh_1_, 20(S)-Rg_2_, 20(S)-Rb_1_, Rb_2_ and Rd in various tissues of different samples were determined by UHPLC-Q/TOF–MS. The results are given in Table 3 and Fig. 5. For most samples (Pq1–5, Pq7–10), the cork contained the most ginsenosides compared with other tissues, with the content ranging from 1094.58 to 269944.16 ng/10^5^ μm^2^. Sometimes, the primary xylem possessed the highest level of ginsenosides (Pq6, Pq11–13), or possessed the second highest level (main root of Pq1, Pq5, Pq7–10), whereas sometimes low ginsenoside level was found in the primary xylem (main root of Pq2–4). The amounts of ginsenosides fluctuated in the cortex. It seemed that if the contents of ginsenosides were low in primary xylem, the contents would be high in cortex (main root of Pq2–4); and if the contents of ginsenosides were high in primary xylem, the cortex would have a medium (main root of Pq1, Pq5, Pq7, Pq8, Pq10) or low (Pq6, Pq9, Pq11–13) level of ginsenosides. The phloem, secondary xylem and cambium usually had fewer ginsenosides than other tissues. For the branch roots of Pq1-4, the cork, xylem and cortex occupied higher contents of ginsenosides than phloem did. Thus, the distribution pattern of ginsenosides in American ginseng was quite distinct from Asian ginseng. Distinctly, the cork, primary xylem or cortex had more ginsenosides than phloem, secondary xylem and cambium in American ginseng. Based on all the above, it was reasonable to deduce that the ratio of total areas of cork, primary xylem and the cortex to the area of whole transection could help to evaluate the quality of American ginsengs.

**Table 3:** Contents of ginsenosides in the tissues from *Panax quinquefolium* samples

Sample no.	Tissue	Amount in unit area (ng/10^5^μm^2^)							
		Rg_1_^a^	Re	Rh_1_	Rg_2_	Rb_1_	Rb_2_	Rd	Sum
Pq1 main root	Cork	67.31	34.58	0.40	8.83	13,247.66	25.51	13.18	13,397.47
	Cortex	18.77	9.50	–^b^	1.83	5576.43	1.02	1.05	5608.60
	Phloem	11.53	7.46	0.25	1.87	4734.50	1.28	1.53	4758.42
	Secondary xylem	9.38	10.74	–	2.11	3176.85	2.20	1.69	3202.97
	Primary xylem	31.12	12.11	0.30	2.07	8104.59	2.15	1.49	8153.83
Pq1 branch root	Cork	74.68	50.67	–	1.16	16,897.70	31.86	7.00	17,063.07
	Cortex	10.84	9.88	–	2.81	7608.60	1.63	3.24	7637.00
	Phloem	5.50	5.46	0.35	2.17	5073.24	0.56	2.72	5090.00
	Xylem	8.10	9.36	–	3.97	7239.49	1.68	12.29	7274.89
Pq2 main root	Cork	130.53	69.38	0.27	3.45	16,012.69	42.06	27.09	16,285.47
	Cortex	36.33	19.63	–	0.75	5244.41	1.16	0.95	5303.23
	Phloem	16.39	8.66	–	2.57	3840.21	1.17	0.72	3869.72
	Secondary xylem	23.34	28.46	–	7.57	2344.29	2.51	1.50	2407.67
	Primary xylem	27.65	29.74	–	4.72	2688.51	3.21	0.93	2754.76
Pq2 branch root	Cork	62.44	46.66	0.30	13.93	17,558.77	52.02	29.52	17,763.64
	Cortex	11.64	8.78	0.36	2.84	3371.72	1.44	2.04	3398.82
	Phloem	11.57	8.17	0.37	4.20	3159.24	1.82	3.55	3188.92
	Xylem	23.09	18.41	0.34	9.31	5805.28	1.48	17.17	5875.08
Pq3 main root	Cork	41.66	18.92	0.39	4.15	269,855	16.80	7.24	269,944.16
	Cortex	18.67	7.19	0.59	1.61	145,606.6	3.61	1.15	145,639.42
	Phloem	11.31	6.40	0.51	0.98	67,598.38	–	1.21	67,618.79
	Secondary xylem	11.69	6.42	0.31	0.84	50,655.09	–	–	50,674.35
	Primary xylem	10.03	5.35	0.33	0.87	19,113.26	0.46	0.30	19,130.60
Pq3 branch root	Cork	23.16	20.68	0.32	4.98	252,865.9	12.32	17.92	252,945.28
	Cortex	6.69	6.20	0.39	2.06	114,430.5	3.50	4.44	114,453.78
	Phloem	5.17	4.43	0.32	1.56	85,663.43	0.95	3.07	85,678.93
	Xylem	6.18	5.95	0.27	0.30	134,882.3	0.70	9.10	134,904.80
Pq4 main root	Cork	48.13	23.62	0.32	0.56	11,972.59	20.01	8.35	12,073.58
	Cortex	11.50	5.10	0.31	0.84	4151.39	1.61	1.43	4172.18
	Phloem	11.85	5.45	0.33	0.52	1685.59	–	1.28	1705.02
	Secondary xylem	9.70	5.35	0.48	0.69	2659.33	1.51	1.45	2678.51
	Primary xylem	6.37	3.43	0.43	0.54	1766.77	0.74	0.47	1778.75
Pq4 branch root	Cork	19.34	20.25	0.30	3.61	20,298.81	19.70	23.67	20,385.68
	Cortex	7.09	7.63	0.40	1.48	12,388.83	5.48	7.41	12,418.32
	Phloem	2.94	4.66	0.32	1.07	5156.83	1.73	9.49	5177.04
	Xylem	7.50	8.57	0.35	2.25	15,479.39	2.95	9.38	15,510.39
Pq5	Cork	1723.58	838.53	10.24	11.41	869.15	167.96	229.08	3849.94
	Cortex	920.69	365.92	4.64	3.35	764.67	11.79	20.06	2091.13
	Phloem	527.99	390.62	2.07	1.50	885.82	6.30	70.71	1884.99
	Secondary xylem	798.60	434.04	0.95	2.28	821.14	6.09	26.04	2089.14
	Primary xylem	1028.47	924.56	1.19	5.86	1365.07	32.65	144.22	3502.02
Pq6	Cork	670.07	34.99	6.03	1.00	582.31	149.25	155.58	1599.24
	Cortex	320.81	13.99	3.79	0.84	364.01	41.22	47.94	792.61
	Phloem	417.83	18.60	2.80	0.94	432.25	5.95	25.73	904.10
	Cambium	605.12	26.43	7.10	1.03	600.16	6.08	40.85	1286.77
	Secondary xylem	906.45	35.99	4.26	0.85	814.07	7.77	59.66	1829.04
	Primary xylem	1501.30	74.73	5.11	1.32	1115.92	23.22	179.22	2900.82
Pq7	Cork	166.40	327.34	1.71	3.93	401.22	66.79	127.19	1094.58
	Cortex	174.18	207.77	1.30	2.98	163.75	16.12	24.81	590.91
	Phloem	119.12	131.49	0.65	1.40	191.36	4.16	21.26	469.43
	Secondary xylem	158.80	110.53	0.60	0.88	157.42	4.33	3.80	436.35
	Primary xylem	187.65	173.03	0.71	0.92	333.41	11.19	30.31	737.22
Pq8	Cork	149.28	1827.33	0.67	12.70	1347.97	41.12	429.50	3808.57
	Cortex	180.35	714.05	0.74	19.35	1173.10	80.68	82.68	2250.96
	Phloem	141.83	732.05	0.56	6.91	1002.23	9.38	49.03	1941.99
	Cambium	144.34	723.85	0.62	9.33	1154.96	5.40	61.17	2099.69
	Secondary xylem	144.52	987.34	0.80	9.24	1478.13	11.02	163.51	2794.55
	Primary xylem	145.17	1302.97	0.95	11.91	1365.79	12.07	218.33	3057.19
Pq9	Cork	907.61	14.06	2.08	0.88	799.16	195.43	170.42	2089.63
	Cortex	160.10	1.99	–	–	179.45	7.47	4.61	353.61
	Phloem	74.54	1.52	–	0.95	60.01	4.97	2.16	144.15
	Secondary xylem	392.43	2.41	–	–	430.97	3.61	22.49	851.91
	Primary xylem	676.25	2.78	0.84	–	1019.24	5.45	69.53	1774.09
Pq10	Cork	668.61	712.57	0.84	6.36	986.29	19.83	67.40	2461.89
	Cortex	139.10	669.75	0.54	14.92	635.65	78.61	39.19	1577.77
	Phloem	123.70	611.79	0.61	6.61	434.33	3.12	14.90	1195.07
	Secondary xylem	146.61	697.48	0.66	5.26	538.81	16.77	19.34	1424.93
	Primary xylem	147.68	743.10	0.62	7.22	714.65	2.12	20.17	1635.56
Pq11	Cork	62.97	537.33	0.88	5.07	511.33	65.37	188.27	1371.23
	Cortex	24.68	320.33	–	3.50	503.92	2.51	36.75	891.69
	Phloem	21.42	344.88	–	3.88	670.87	2.04	83.75	1126.83
	Secondary xylem	8.58	340.35	–	3.60	564.94	2.98	159.17	1079.62
	Primary xylem	22.13	619.29	0.56	7.31	916.71	3.81	364.94	1934.75
Pq12	Cork	115.07	518.18	1.85	6.69	634.75	104.45	319.73	1700.73
	Cortex	67.05	342.52	0.55	3.34	560.80	9.01	43.48	1026.75
	Phloem	69.79	375.80	–	3.56	871.87	3.74	48.98	1373.75
	Secondary xylem	45.97	610.82	0.61	4.39	1117.76	6.46	211.26	1997.28
	Primary xylem	147.28	871.49	0.47	5.95	1021.44	9.45	132.19	2188.28
Pq13	Cork	82.82	766.07	0.92	22.68	568.79	88.17	125.20	1654.66
	Cortex	33.11	428.44	–	6.46	453.02	7.45	37.26	965.74
	Phloem	34.04	547.62	–	5.32	526.29	3.31	41.08	1157.65
	Secondary xylem	41.02	453.95	0.52	8.36	772.36	5.45	104.84	1386.50
	Primary xylem	37.32	893.53	–	14.27	922.80	3.81	166.35	2038.07

^a^Ginsenoside

^b^Under detection limit

It was reported that the outer part of the *P. quinquefolium* root contained more ginsenosides than the center part [25]. However, another paper found that the peak areas of ginsenosides in the center part were larger than those of the outer part [26]. The outer part includes the cork and cortex, while the center part represented the primary xylem for most samples or xylem for branch roots. Our research illustrated that the both situations existed simultaneously in American ginseng.

Although *P. quinquefolium* and *P. ginseng* were closely related species which contained many common saponin constituents, their distribution patterns of ginsenosides were quite different. The most obvious difference was that the ginsenosides were not only concentrated in the cork and cortex, but also inclined to be accumulated in the primary xylem in American ginseng. This was identical with the morphological and microscopical characteristics of Asian and American ginseng. In detail, American ginseng was harder than Asian ginseng, and was more difficult to be broken. At the same time, under the fluorescence microscope, it was found that xylem of American ginseng usually differentiated into primary and secondary xylem, while the differentiation was scarely seen in the xylem of Asian ginseng. That is to say that the developed primary xylem was absent in Asian ginseng. The different microscopic structures between American ginseng and Asian ginseng may explain their distinct distribution patterns of ginsenosides in various tissues..

Such similar phenomenon was also found in Bupleuri Radix material. *Bupleurum chinense* DC. and *B. scorzoneri folium* Willd. were both original plants of Bupleuri Radix in China. Meanwhile, *B. falcatum* L. was recorded by Japanese Pharmacopoeia as the original plant of Bupleuri Radix. Recent research found that although saikosaponins were mostly distributed in the cork and cortex in the three species, the cork of *B. scorzoneri folium* and *B. falcatum* contained more saikosaponin a, c, d than the cortex, while the opposite situation was found in *B. chinense* [7]. Thus, the phenomenon that related plants had different distribution patterns of the same secondary metabolites was not an accident. The exact mechanism causing the phenomenon deserved to be further explored.

## Conclusion

In conclusion, LMD, fluorescence microscopy, and UHPLC-Q/TOF–MS were applied to profile and determine tissue-specific chemicals of *P. quinquefolium* in this study. As a result, the cork, primary xylem or cortex had more ginsenosides than phloem, secondary xylem and cambium in American ginseng. Thus, the ratio of total areas of cork, primary xylem and the cortex to the area of the whole transection showed a potential to be used as a reference to judge the quality of American ginsengs.

## Experimental

### Plant material

As seen from Table 1 and Fig. 1, four fresh *P. quinquefolium* samples (Pq1–4) were collected from Mulin County, Mudanjiang City, Heilongjiang Province, China. Nine dried samples (Pq5–13) of various commercial types were purchased from Hong Kong herbal markets. All of them were identified by Dr. Zhitao Liang from the School of Chinese Medicine, Hong Kong Baptist University. The voucher specimens were deposited in the Bank of China (Hong Kong) Chinese Medicines Centre of Hong Kong Baptist University. Collected samples were stored at −20 °C before use.

### Chemicals and reagents

Chemical standards of ginsenosides Rg_1_, 20(*S*)-Rg_2_, Re, 20(*S*)-Rh_1_, Rb_1_, Rb_2_ and Rd were purchased from Shanghai Tauto Biotech Company (Shanghai, China). Acetonitrile and methanol of HPLC grade were from E. Merck (Darmstadt, Germany), and formic acid of HPLC grade was from Tedia (Fairfield, USA). Water was prepared by a Milli-Q system (Millipore, Bedford, MA, USA).

### Laser microdissection and sample solution preparations

The dried materials were firstly softened by infiltrating with water-soaked-non-cellulose paper before frozen section. The softened and fresh roots were cut into small sections, embedded in cryomatrixTM (Thermo Shandon Limited, U.K.), and then placed on a cutting platform in the cryobar of a cryostat (Thermo Shandon As620 Cryotome, U.K.) at −20 °C. Serial slices of 40 μm in thickness were cut at −10 °C. Each sectioned tissue slice was mounted directly to a non-fluorescent PET microscope steel frame slide (76 mm × 26 mm, 1.4 μm thick, Leica Microsystems, Germany). The slide was observed with a Leica LMD 7000 microscope system (Leica, Benshein, Germany) in fluorescence mode with a dichromatic mirror. Microdissection was conducted by a DPSS laser beam at 349 nm wavelength, aperture of 12, speed of 10, power of 50–60 μJ and pulse frequency of 2895 Hz under a Leica LMD-BGR fluorescence filter system at 10x magnification. Tissue parts within an area of approximately 1 × 10^6^ μm^2^ were determined as the investigated size and dissected separately under fluorescence inspection mode. The microdissected tissues fell into caps of 500 μL microcentrifuge tubes (Leica, Germany) by gravity.

The separated tissue part in each cap was transferred to the bottom of the tube through centrifugation (Centrifuge 5415R, Eppendorf, Hamburg, Germany) at 12,000 rpm for 5 min. 100 μL methanol was added into each microcentrifuge tube. The tube was sonicated for 30 min (CREST 1875HTAG ultrasonic processor, USA). The microcentrifuge tube was centrifuged again for 10 min at 12,000 rpm, and 4 °C. 90 μL of the supernatant was transferred to a glass insert with plastic bottom spring (400 μL, Grace, USA) in a 1.5 mL brown HPLC vial (Grace, USA) and stored at 4 °C for analysis.

### Qualitative and quantitative analysis

UHPLC-QTOF–MS analysis was performed on an Agilent 6540 ultra-high definition accurate mass quadrupole time-of-flight spectrometer with UHPLC (UHPLC-QTOF–MS, Agilent Technologies, USA). A UPLC C_18_ analytical column (2.1 mm × 100 mm, I.D. 1.7 μm, ACQUITY UPLC^®^ BEH, Waters, USA) was used for separation, coupled with a C_18_ pre-column (2.1 mm × 5 mm, I.D. 1.7 μm, VanGuardTM BEH, Waters, USA) at room temperature of 20 °C. The mobile phase was a mixture of water (A) and acetonitrile (B), both containing 0.1 % formic acid, with an optimized linear gradient elution as follows: 0–3 min, 10–20 % B; 3–25 min, 20–38 % B; 25–30 min, 38–85 % B; 30–30.1 min, 85–100 % B; 30.1–32 min, 100 % B; 32–32.1 min 100–10 % B with 4 min of balance. The injection volume was 3 μL for tissue sample. The flow rate was set at 0.35 mL/min. The mass spectra were acquired in negative mode by scanning from 100 to 1700 in mass to charge ratio (*m*/*z*). The MS analysis was performed under the following operation parameters: dry gas temperature 300 °C, dry gas (N_2_) flow rate 8 L/min, nebulizer pressure 45 psi, Vcap 3000, nozzle voltage 500 V, and fragmentor voltage 180 V. The energies for collision-induced dissociation (CID) were set at 30 and 45 eV respectively for the fragmentation information.

Data analysis was performed with Agilent MassHunter Workstation software-Qualitative Analysis and Q-TOF Quantitative Analysis (version B.04.00, Build 4.0.479.5, Service Pack 3, Agilent Technologies, Inc. 2011). By searching databases including PubMed of the US National Library Medicine and the National Institutes of Health, Scifinder Scholar of American Chemical Society and Chinese National Knowledge Infrastructure (CNKI) of Tsinghua University, all chemicals reported in the literatures as derived from *Panax* species were summarized in a Microsoft Office Excel table to establish a database, which includes the name, molecular formula, and molecular weight of each chemical. The “Search Database” in the “Identify Compounds” in Agilent MassHunter Workstation software-Qualitative Analysis was used to identify the chromatographic peaks.

To semi-quantitatively determine the spatial distributions of the individual metabolites in different tissue regions, the contents of chemical markers including ginsenosides Rg_1_, 20(S)-Rg_2_, Re, 20(S)-Rh_1_, Rb_1_, Rb_2_ and Rd in various microdissected tissues were relatively determined using the above UHPLC-QTOF–MS method. Linearity was examined within selected concentration range with different levels and applied to calculate the amounts of these analytes in tissue extracts.

## Abbreviations

LMD: laser microdissection
UHPLC-Q/TOF-MS: ultra-high performance liquid chromatography-quadrupole/time-of-flight- mass spectrometry
